# The prevalence patterns and risk factor profiles of poor muscle health and its associated components in multiethnic older Asians: The PIONEER study

**DOI:** 10.1002/jcsm.13483

**Published:** 2024-04-22

**Authors:** Preeti Gupta, Tai‐Anh Vu, Ryan E.K. Man, Eva K. Fenwick, Laura Tay, Ng Yee Sien, David Ng, Koh Hong Xiang Frederick, Eu‐Leong Yong, Samuel T.H. Chew, Ecosse L. Lamoureux

**Affiliations:** ^1^ Singapore Eye Research Institute Singapore National Eye Centre Singapore; ^2^ The Ophthalmology and Visual Sciences Academic Clinical Programme (EYE ACP) Duke‐NUS Medical School Singapore; ^3^ Department of Geriatric Medicine Sengkang General Hospital Singapore; ^4^ Department of Rehabilitation Medicine, Division of Medicine Singapore General Hospital Singapore; ^5^ Department of Nuclear Medicine and Molecular Imaging Singapore General Hospital Singapore; ^6^ Department of Surgery Sengkang General Hospital Singapore; ^7^ Department of Obstetrics and Gynecology National University of Singapore Singapore; ^8^ Department of Geriatric Medicine Changi General Hospital Singapore; ^9^ SingHealth Duke‐NUS Medicine Academic Clinical Programme Singapore; ^10^ Yong Loo Lin School of Medicine National University of Singapore Singapore; ^11^ The University of Melbourne Australia

**Keywords:** Muscle function, Muscle health, Muscle mass, Muscle strength, Physical performance, Sarcopenia

## Abstract

**Background:**

We aim to determine the multiethnic patterns of the prevalence and associated factors of poor muscle health and its associated components in older Chinese, Malays, and Indian Asian adults.

**Methods:**

We included 2199 participants (mean age ± SD: 72.9 ± 8.3 years; 54.3% female) from the baseline assessment of the Population Health and Eye Disease Profile in Elderly Singaporeans (PIONEER; 2017–2022) cohort study. Poor muscle health was defined as the presence of either low muscle mass (DEXA), or low muscle strength (handgrip strength), or low physical performance (gait speed). Its components include poor muscle function (low muscle strength and/or low physical performance without low muscle mass), pre‐sarcopenia (low muscle mass only), and any sarcopenia (low muscle mass with low muscle strength and/or low physical performance). Sociodemographic, clinical, and lifestyle factors were assessed using biochemistry, clinical tests, and validated questionnaires. Regression models were utilized to evaluate the independent risk factors of poor muscle health and its components.

**Results:**

The national census‐adjusted prevalence of poor muscle health (88%) was similar across the three ethnic groups. However, Chinese individuals had higher prevalence of pre‐sarcopenia and any sarcopenia, and a lower prevalence of poor muscle function compared with Indians or Malays. We observed ethnic differences in modifiable risk factors (low physical activity, diabetes, osteoporosis, and obesity) of poor muscle health and its components. Although obesity was protective of pre‐sarcopenia (RRR = 0.19, 95% CI: 0.11, 0.36) and any sarcopenia (RRR = 0.29, 95% CI: 0.18, 0.47) in the overall population and across ethnic groups, it was associated with 1.7 times (95% CI: 1.07, 2.67) the likelihood of poor muscle function in the entire population.

**Conclusions:**

Almost 90% of community dwelling Singaporean aged ≥60 years have poor muscle health across the three ethnic groups with ethnic disparities in modifiable risk factors, highlighting an urgent need for community‐wide targeted interventions to promote muscle health.

## Introduction

An estimated 1.5 billion people will be aged ≥60 years by 2030.^1^, ^2^ Thus, interest on muscle health has surged, as it has grave physiological and clinical repercussions in older adults.^3^, ^4^ Normal muscle health can be broadly defined as adequate muscle mass and function (muscle strength and physical performance).^3^, ^5^ Hence, poor muscle health may be defined as the presence of either low muscle mass, low muscle strength, or low physical performance. In this study, we further categorize poor muscle health into its components, including pre‐sarcopenia (poor muscle mass alone), poor muscle function (low muscle strength and/or low physical performance without low muscle mass), and any sarcopenia in which both muscle mass and function are affected.

Among the components of poor muscle health, sarcopenia have been well‐recognized due to the objective loss of muscle mass contributing to their dire consequences.^6^ Nonetheless, previous research has shown that poor muscle function may occur without any muscle mass loss because muscular function is contributed by neuromuscular and hormonal crosstalks other than muscle quantity.^7^ As such, the stratification of poor muscle health by its components is important because early losses of muscle mass or muscle function alone may also predict poor clinical outcomes.^8^, ^9^ Critically, no studies have comprehensively evaluated the prevalence and risk factors of poor muscle health and its components (pre‐sarcopenia, poor muscle function, and any sarcopenia) in a representative community‐dwelling multiethnic Asian population with diverse cultural, and life‐style behaviour.^10^, ^11^ Addressing this critical knowledge gap may lead to the implementation of early interventions to promote muscle health as part of the new paradigm of older adults having healthy, independent and meaningful lives.^12^

Using a nationwide representative sample of multiethnic older adults (Chinese, Indians, and Malays) living across Singapore from The PopulatIoN Health and Eye Disease Profile (PIONEER) study, we determined the overall‐, age‐, and ethnic‐stratified prevalence and associated factors of poor muscle health and its components (pre‐sarcopenia, poor muscle function, and any sarcopenia). We hypothesize that poor muscle health and its components are common among older adults, with different prevalence across ethnic groups, and more likely to occur with increasing age. Furthermore, we expect that several sociodemographic, clinical and lifestyle factors will be associated with poor muscle health and its components, and that the differences in lifestyle, nutritional habits, culture (e.g., differences in health‐seeking behaviour),^13^ and genetic predisposition may potentially contribute to the disparate ethnic‐specific risk factor profile.

## Methods

### Study population and design

PIONEER is a population‐based cohort study that aims to evaluate the epidemiology; and patient‐centred and economic impact of age‐related sensory loss, together with its overarching relationship with systemic aging. The baseline visit was conducted between 2017 and 2022 among Chinese, Malay, and Indian adults aged ≥60 years living in Singapore. A detailed methodology is reported elsewhere,^14^ and briefly in the supporting information. The study protocol followed the Declaration of Helsinki, and ethics approval from Singapore's centralized institutional review board was obtained before the study began recruitment (#2016/3089).

### Assessment of the components of muscle health

Muscle Mass was assessed by a trained and certified radiographer performed dual energy X‐ray absorptiometry (DXA; Discovery‐W. Hologic Inc. Bedford‐MA) to measure whole and regional body compositions including, fat and muscle mass, and bone mineral density (BMD; of the femoral neck, total femur, and lumbar spine). Analyses were performed using Hologic Horizon W software (version 5) in its default configuration. *Low muscle mass* was defined as appendicular‐lean‐mass/height^2^ of <7 kg/m^2^ for males and <5.4 kg/m^2^ for females.^6^

Hand grip strength was assessed using the Jamar Plus+ Digital Hand Dynamometer. Participants' grip strength (in kg) in the dominant hand was measured three times, with the participant seated and elbow flexed at 90 degrees, with a rest period of 30 s between each measurement. Participants were asked to provide maximum effort for each measure and the maximal reading was utilized in analyses. If the participants were unable to perform measurement in the dominant hand due to health reasons (swelling, inflammation, pain, or injury), measurements were taken using the non‐dominant hand. *Low muscle strength* was defined as a maximal grip strength <28 kg and <18 kg, in men and women, respectively.^6^

Gait speed was assessed by measuring the participant's usual gait (in m/s) over a 4 m course, and timing (recorded in seconds) was stopped when the first foot completely crossed the 4 m mark. *Low physical performance* was defined as gait speed of <1.0 m/s.^6^

Additionally, *fat mass index* (*FMI*) was calculated by dividing the individual's total body fat mass (kg), as measured by DXA, by their height (m) squared. The recent Yishun study conducted in Singapore found FMI as the most preferred measure for adiposity. Thus, we defined obesity as FMI >7.63 kg/m^2^ for men and >9.93 kg/m^2^ for women, based on the sex‐specific upper two quintiles of the Yishun study population.^15^

### Definitions of poor muscle health and components

The presence of any one low muscle mass, low muscle strength, or low physical performance was defined as *poor muscle health*. The subcategories of poor muscle health include poor muscle function, pre‐sarcopenia, and any sarcopenia. *Poor muscle function* was defined as low muscle strength and/or low physical performance but without low muscle mass. Poor muscle function comprises three groups: low muscle strength only, low physical performance only, and both low muscle strength and low physical performance. *Pre‐sarcopenia* was defined as the occurrence of low muscle mass only.^16^ Low muscle mass with the presence of either low muscle strength or low gait speed was considered *sarcopenia* whereas the presence of all three factors constituted *severe* sarcopenia.^6^ *Any sarcopenia* was defined as the presence of either sarcopenia or severe sarcopenia.

### Statistical analyses

All analyses were conducted using Stata 17.0 (StataCorp LLC, TX, USA). In descriptive analyses, we compared baseline sociodemographic and clinical characteristics across three ethnic groups using analysis of variance for continuous variables and the chi‐squared tests for categorical variables. As we oversampled minority races, female, and older participants, the prevalence of poor muscle health and its components [poor muscle function (low muscle strength only, low physical performance only, and both low muscle strength and low physical performance), pre‐sarcopenia, and any sarcopenia (sarcopenia and severe sarcopenia)] for the entire sample and after being stratified by age and ethnicity were determined by weighting individuals according to their sampling probabilities and standardizing to the 2020 Singapore's population census. The percentages have been independently adjusted based on the census of 2020, and hence cannot be derived from the unadjusted (*n*) in each group.

Multivariable logistic regression (odds ratio [OR]) was used to assess the associations between sociodemographic, clinical and lifestyle factors (age, sex, ethnicity, low SES, living alone, obesity, smoking status, weekly alcohol consumption, caloric intake, low PA, polypharmacy, diabetes, hypertension, IHD, stroke, CKD, osteoporosis, and high CRP level; see Data S1 for details of these variables) with poor muscle health in the overall sample and respective ethnic groups. To investigate the associations between the above factors and components of muscle health, multinomial logistic regression model [relative risk ratio (RRR)] was performed with the outcome being categorized into none, pre‐sarcopenia, poor muscle function, and any sarcopenia. All regression estimates were reported with 95% confidence intervals (CI) and statistical significance was concluded at a *P*‐value <0.05.

## Results

Of the 2643 enrolled study participants, 2 were <60 years old, 5 of races other than Chinese, Malay and Indian, and 437 had missing data necessary for muscle health categorization, leaving 2199 participants for the final analyses.

### Characteristics of participants

Of the 2199 participants (mean age ± SD 72.9 ± 8.3 years), 1105 (50.25%), 580 (26.38%), and 514 (23.37%) were of Chinese, Malay, and Indian ethnicities, respectively, and 1187 (54.0%) were female (Table 1). Compared with Chinese, Malays and Indians had significantly lower SES, poorer systemic profile including, higher CRP, obesity, DM, HTN, IHD, CKD, and polypharmacy, a higher proportion of them were current smokers and alcohol consumers, were physically less active, and had lower caloric intake. Contrarily, Chinese had significantly higher prevalence of osteoporosis (40%) than Malays (~30%) and Indians (~25%).

**Table 1:** Demographic, systemic, and socioeconomic characteristics of participants in the PIONEER study

	Overall (*N* = 2199)	Chinese (*N* = 1105)	Malay (*N* = 580)	Indian (*N* = 514)	*P*‐value^a^
Age (year)	72.9 (8.3)	73.4 (8.2)	71.6 (8.1)	73.1 (8.5)	<0.001
Age					<0.001
60–69	873 (39.7)	392 (25.5)	272 (46.9)	209 (40.7)	
70–79	732 (33.3)	399 (36.1)	173 (29.8)	160 (31.1)	
≥80	594 (27.0)	314 (28.4)	135 (23.3)	145 (28.2)	
Sex (female)	1187 (54.0)	592 (53.6)	308 (53.1)	287 (55.8)	0.62
Low socioeconomic status	104 (5.0)	41 (3.9)	39 (4.0)	24 (4.9)	0.025
Living alone	205 (9.6)	110 (10.3)	47 (8.3)	48 (9.6)	0.42
High C‐reactive protein	593 (30.4)	195 (20.7)	198 (36.5)	200 (43.0)	<0.001
Obesity	875 (39.8)	269 (24.3)	326 (56.2)	280 (54.5)	<0.001
Osteoporosis	736 (33.5)	436 (39.5)	172 (29.7)	128 (25.1)	<0.001
Diabetes	747 (37.3)	287 (29.6)	224 (40.5)	236 (49.1)	<0.001
Hypertension	1876 (85.5)	916 (83.3)	504 (87.1)	456 (88.7)	0.007
Ischaemic heart disease	319 (17.1)	132 (14.0)	74 (14.9)	113 (26.7)	<0.001
Stroke	81 (4.3)	38 (3.9)	19 (3.7)	24 (5.5)	0.31
Chronic kidney disease	416 (20.9)	162 (16.6)	151 (27.6)	103 (21.8)	<0.001
Polypharmacy	440 (20.5)	176 (16.3)	127 (22.8)	137 (27.4)	<0.001
Current smoker	187 (8.8)	64 (6.0)	88 (15.5)	35 (7.0)	<0.001
Alcohol frequency					<0.001
None	1858 (89.9)	884 (86.3)	564 (99.3)	410 (86.5)	
≤4 days per week	155 (7.5)	103 (10.1)	2 (0.4)	50 (10.6)	
>4 days per week	53 (2.6)	37 (3.6)	2 (0.4)	14 (3.0)	
Low physical activity	1240 (61.5)	550 (54.9)	361 (65.4)	329 (71.2)	<0.001
Low caloric intake	825 (48.4)	425 (48.3)	188 (43.5)	212 (54.2)	0.009

Data are represented as mean (SD) or number (%).

^a^ ANOVA or chi‐squared test.

### Prevalence of presence of poor muscle health and its components stratified by age and ethnicity

The national census‐adjusted prevalence of poor muscle health was 88.4% (*n* = 2016) but was not significantly different between ethnic groups (88.2%, 89.6%, and 89.6% in Chinese, Malays, and Indians, respectively). The prevalence of poor muscle health and its components increased with increasing age consistently across ethnic groups (Table 2). Among older adults with poor muscle health, 27.8% (*n* = 749), 16.6% (*n* = 223), 44.0% (*n* = 1044) had poor muscle function, pre‐sarcopenia, and any sarcopenia, respectively. There were higher prevalence of pre‐sarcopenia and any sarcopenia and lower prevalence of poor muscle function in Chinese, compared with Indians or Malays (Table 2).

**Table 2:** Prevalence of poor muscle health and its components stratified by age and ethnicity in the PIONEER study

Age group (year)	All (*N* = 2199), *n* (%)	Ethnicity		
		Chinese (*N* = 1105), *n* (%)	Malay (*N* = 580), *n* (%)	Indian (*N* = 514), *n* (%)
**Poor muscle health**				
60–69	731 (82.8)	322 (82.2)	234 (86.0)	175 (83.6)
70–79	695 (94.3)	375 (94.0)	164 (95.0)	156 (97.6)
≥80	590 (99.1)	311 (98.9)	135 (100)	144 (99.4)
*P*‐trend	<0.001	<0.001	<0.001	<0.001
Total	2016 (88.4)	1008 (88.2)	533 (89.6)	475 (89.6)
**Poor muscle function**				
60–69	330 (28.4)	92 (23.5)^a^	159 (57.2)^d^	79 (37.3)
70–79	261 (29.2)	106 (26.1)^a^	87 (51.6)	68 (42.1)
≥80	158 (22.5)	58 (20.4)^a^	53 (41.4)^d^	47 (29.7)
*P*‐trend	0.17	0.75	0.009	0.57
Total	749 (27.8)	256 (23.8)^a^	299 (54.2)^d^	194 (37.7)
**Pre‐sarcopenia**				
60–69	151 (23.1)	102 (26.1)^b^	20 (7.7)^e^	29 (14.0)
70–79	62 (10.5)	46 (11.5)^c^	4 (2.1)^e^	12 (7.7)
≥80	10 (2.4)	10 (2.8)	0 (0)	0 (0)
*P*‐trend	<0.001	<0.001	<0.001	<0.001
Total	223 (16.6)	158 (18.4)^b^	24 (5.5)^e^	41 (10.7)
**Any sarcopenia**				
60–69	250 (31.3)	128 (32.7)^c^	55 (21.1)^e^	67 (32.2)
70–79	372 (54.6)	223 (56.4)^c^	73 (41.3)	76 (47.8)
≥80	422 (74.1)	243 (75.7)^c^	82 (58.6)	97 (69.7)
Total	<0.001	<0.001	<0.001	<0.001
*P*‐trend	1044 (44.0)	594 (46.0)^b^	210 (29.9)^e^	240 (40.8)

Data are presented as number of participants (weighted prevalence %). Weighted prevalence was calculated with sampling weights specific to each age group, gender, and ethnicity to adjust for oversampling and post‐stratification weights to align to the population distribution based on the 2020 Singapore Census.

^a^ Significantly lower than Malay and Indian.

^b^ Significantly higher than Malay and Indian.

^c^ Significantly higher than Malay only.

^d^ Significantly higher than Indian.

^e^ Significantly lower than Indian.

**Figure 1:**
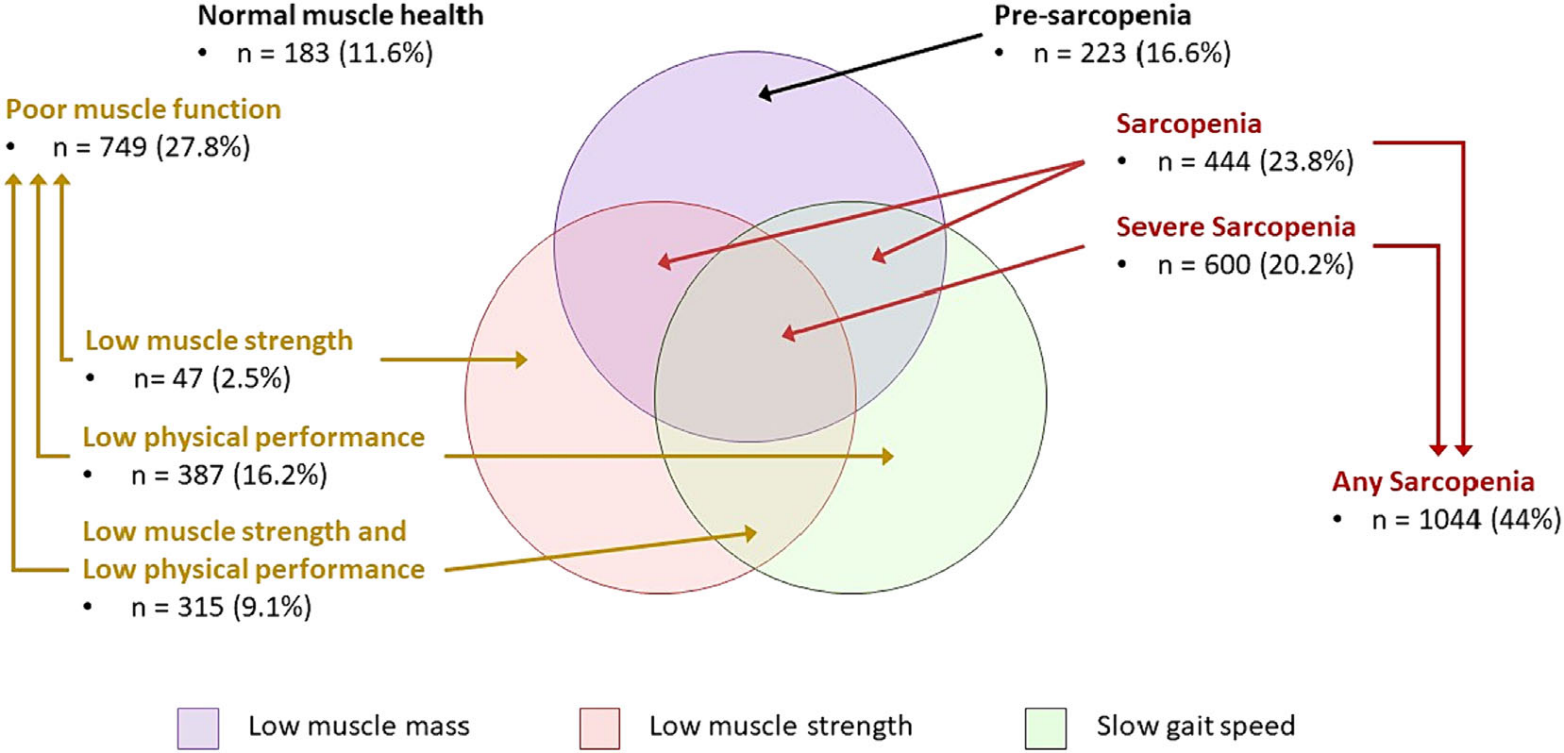
National census‐adjusted prevalence of poor muscle health and its components. The percentages have been independently adjusted based on the census of 2020, and hence cannot be derived from the unadjusted (*n*) in each group. *Normal muscle health*: absence of all three factors (low muscle mass, low muscle strength, and low physical performance); *poor muscle health*: the presence of any one low muscle mass, low muscle strength, or low physical performance; *poor muscle function*: low muscle strength and/or low physical performance but without low muscle mass. Poor muscle function comprises three groups: low muscle strength only, low physical performance only, and both low muscle strength and low physical performance. *Pre‐sarcopenia:* the occurrence of low muscle mass only; *sarcopenia*: low muscle mass with the presence of either low muscle strength or low gait speed, whereas the presence of all three factors constituted *severe sarcopenia*. *Any sarcopenia*: the presence of either sarcopenia or severe sarcopenia.

Among individuals with poor muscle function, 2.5% (*n* = 47), 16.2% (*n* = 387), and 9.1% (*n* = 315) had low grip strength only, low physical performance only, and both low muscle strength and low physical performance, respectively. For those with any sarcopenia, 23.8% (*n* = 444) and 20.2% (*n* = 600) experienced sarcopenia and severe sarcopenia (Figure 1).

### Factors associated with poor muscle health

In multivariable models, older age (per year increase: odds ratio [OR]: 1.16, 95% CI: 1.11, 1.21; *P* < 0.001), female gender (OR: 2.22; 95% CI: 1.43, 3.45; *P* < 0.001), presence of osteoporosis (OR: 2.55, 95% CI: 1.42, 4.56; *P* = 0.002), diabetes (OR: 1.67, 95% CI: 1.04, 2.70; *P* = 0.035), and low PA (OR: 1.57, 95% CI: 1.06, 2.33; *P* = 0.025) were associated with higher odds of poor muscle health (Table 3). Conversely, obesity (OR: 0.59; 95% CI: 0.39, 0.91; *P* = 0.017) was associated with significantly lower odds of poor muscle health.

**Table 3:** Factors associated with poor muscle health in the PIONEER study

	Total (*N* = 1362)		Chinese (*N* = 679)		Malay (*N* = 347)		Indian (*N* = 278)	
Risk factors	OR (95% CI)^a^	*P*‐value	OR (95% CI)^b^	*P*‐value	OR (95% CI)^b^	*P*‐value	OR (95% CI)^b^	*P*‐value
Age	1.16 (1.11, 1.21)	<0.001	1.17 (1.11, 1.24)	<0.001	1.09 (1.01, 1.18)	0.024	1.19 (1.08, 1.31)	0.001
Sex (female)	2.22 (1.43, 3.45)	<0.001	2.91 (1.59, 5.32)	0.001	1.07 (0.42, 2.72)	0.89	1.36 (0.53, 3.47)	0.52
Race								
Chinese	Reference		‐	‐	‐	‐	‐	‐
Malay	1.58 (0.95, 2.61)	0.078	‐	‐	‐	‐	‐	‐
Indian	1.37 (0.81, 2.32)	0.25	‐	‐	‐	‐	‐	‐
Low socioeconomic status	3.39 (0.43, 26.90)	0.25	‐	‐	‐	‐	‐	‐
Living alone	1.74 (0.67, 4.50)	0.25	1.72 (0.48, 6.16)	0.4	2.77 (0.35, 21.97)	0.33	1.45 (1.75, 12.06)	0.73
High C‐reactive protein	0.98 (0.63, 1.52)	0.91	1.19 (0.60, 2.37)	0.61	0.81 (0.38, 1.72)	0.58	1.13 (0.44, 2.95)	0.8
Obesity	0.59 (0.39, 0.91)	0.017	0.50 (0.28, 0.91)	0.024	0.60 (0.26, 1.40)	0.24	0.95 (0.38, 2.34)	0.91
Osteoporosis	2.55 (1.42, 4.56)	0.002	3.18 (1.48, 6.82)	0.003	1.66 (0.58, 4.70)	0.95	3.10 (0.66, 14.42)	0.15
Diabetes	1.67 (1.04, 2.70)	0.035	2.51 (1.1, 5.54)	0.022	0.89 (0.38, 2.07)	0.78	1.26 (0.52, 3.06)	0.61
Hypertension	0.86 (0.53, 1.40)	0.54	1.06 (0.56, 2.02)	0.86	1.94 (0.79, 4.72)	0.15	0.11 (0.01, 0.89)	0.038
Ischaemic heart disease	1.26 (0.63, 2.55)	0.51	1.34 (0.47, 4.03)	0.6	1.49 (0.30, 7.27)	0.62	1.45 (0.48, 4.38)	0.51
Stroke	1.31 (0.36, 4.70)	0.68	‐	‐	‐	‐	‐	‐
Chronic kidney disease	0.61 (0.32, 1.19)	0.15	0.38 (0.16, 0.90)	0.027	0.82 (0.26, 2.59)	0.74	2.06 (0.24, 17.76)	0.51
Polypharmacy	1.32 (0.66, 2.62)	0.084	0.84 (0.29, 2.41)	0.75	2.93 (0.76, 11.33)	0.12	1.28 (0.37, 4.37)	0.7
Current smoker	0.86 (0.46, 1.61)	0.64	0.71 (0.29, 1.75)	0.45	1.15 (0.35, 3.76)	0.57	0.81 (0.21, 3.00)	0.75
Alcohol frequency								
None	Reference		‐	‐	‐	‐	‐	‐
≤4 days per week	0.68 (0.38, 1.19)	0.18	‐	‐	‐	‐	‐	‐
>4 days per week	3.33 (0.76, 14.63)	0.11	‐	‐	‐	‐	‐	‐
Low physical activity	1.57 (1.06, 2.33)	0.025	1.86 (1.07, 3.22)	0.028	1.24 (0.58, 2.67)	0.57	2.21 (0.96, 5.10)	0.063
Low caloric intake	0.98 (0.65, 1.49)	0.93	1.21 (0.68, 2.15)	0.52	0.93 (0.41, 2.10)	0.86	0.98 (0.40, 2.40)	0.97

Abbreviations: CI, confidence interval; OR, odds ratio.

^a^ Adjusted for age, sex, ethnicity, low socioeconomic status, living alone, high C‐reactive protein level, obesity, presence of systemic comorbidities (diabetes, hypertension, ischaemic heart disease, stroke, chronic kidney disease, and osteoporosis), polypharmacy, smoking status, weekly alcohol consumption, low physical activity, and low caloric intake.

^b^ Adjusted for age, sex, living alone, high C‐reactive protein level, obesity, presence of systemic comorbidities (diabetes, hypertension, ischaemic heart disease, chronic kidney disease, osteoporosis), polypharmacy, smoking status, low physical activity, and low caloric intake. Low socioeconomic status, stroke, and alcohol were dropped from the model due to low cell count.

### Factors associated with poor muscle health stratified by ethnicity

Of the above factors, only age was consistently significant across the three ethnic groups. In Chinese, all the above factors remained significant; moreover, CKD (OR: 0.38, 95% CI: 0.16, 0.90; *P* = 0.027) was additionally associated with lower odds of poor muscle health. None of the above factors retained their significance in Malays. For Indians, only low PA was associated with higher odds while hypertension was associated with lower odds (OR: 0.11, 95% CI: 0.01, 0.89; *P* = 0.038) of poor muscle health (Table 3).

### Factors associated with components of poor muscle health

For factors associated with components of poor muscle health (Table 4) in the overall population: older age was associated with a higher risk of poor muscle function (per year increase: RRR: 1.14, 95% CI: 1.10, 1.19; *P* < 0.001) and any sarcopenia (per year increase: RRR: 1.23, 95% CI: 1.18, 1.28; *P* < 0.049), and female gender was a risk factor for poor muscle function (RRR: 1.73; 95% CI: 1.00, 2.99; *P* < 0.001) and pre‐sarcopenia (RRR: 3.33; 95% CI: 2.07, 5.38; *P* < 0.001). Although obesity was protective of pre‐sarcopenia (RRR = 0.19, 95% CI: 0.11, 0.36) and any sarcopenia (RRR = 0.29, 95% CI: 0.18, 0.47), it was associated with 1.7 times (95% CI: 1.07, 2.67) the likelihood of poor muscle function. The presence of osteoporosis was a risk factor for all three components (RRR: 1.79, 95% CI: 0.96, 3.33; *P* = 0.065 for poor muscle function; RRR: 2.33, 95% CI: 1.21, 4.51; *P* = 0.012 for pre‐sarcopenia; RRR: 3.33, 95% CI: 1.82, 6.09; *P* < 0.001 for any sarcopenia), while diabetes (RRR: 2.13, 95% CI: 1.28, 3.53; *P* = 0.003 for poor muscle function; RRR: 1.68, 95% CI: 1.00, 2.82; *P* = 0.05 for any sarcopenia) and low PA (RRR: 1.55, 95% CI: 1.01, 2.38; *P* = 0.043 for poor muscle function; RRR: 1.76, 95% CI: 1.41, 2.71; *P* = 0.011 for any sarcopenia) were associated with approximately twice the risk of poor muscle function and any sarcopenia. Interestingly, higher consumption of alcohol was associated with five times (95% CI: 1.12, 24.46; *P* = 0.043) the likelihood of poor muscle function only.

**Table 4:** Factors associated with components of poor muscle health in the PIONEER study

	Pre‐sarcopenia		Poor muscle function		Any sarcopenia	
Risk factors	RRR (95% CI)	*P*‐value	RRR (95% CI)	*P*‐value	RRR (95% CI)	*P*‐value
Age	1.05 (1.00, 1.10)	0.061	1.14 (1.10, 1.19)	<0.001	1.23 (1.18, 1.28)	<0.001
Sex (female)	1.73 (1.00, 2.99)	0.049	3.33 (2.07, 5.38)	<0.001	1.48 (0.92, 2.40)	0.11
Race						
Chinese	Reference		Reference		Reference	
Malay	0.57 (0.29, 1.14)	0.11	2.50 (1.47, 4.27)	0.001	1.22 (0.71, 2.12)	0.48
Indian	1.33 (0.69, 2.55)	0.39	1.57 (0.89, 2.77)	0.12	1.38 (0.77, 2.44)	0.28
Low socioeconomic status	3.20 (0.32, 31.48)	0.32	3.19 (0.38, 26.52)	0.28	3.55 (0.43, 29.04)	0.24
Living alone	1.44 (0.47, 4.41)	0.52	1.71 (0.64, 4.58)	0.28	1.86 (0.69, 5.00)	0.22
High C‐reactive protein	0.88 (0.49, 1.56)	0.65	0.91 (0.57, 1.46)	0.7	1.07 (0.66, 1.74)	0.79
Obesity	0.19 (0.11. 0.36)	<0.001	1.69 (1.07, 2.67)	0.025	0.29 (0.18, 0.47)	<0.001
Osteoporosis	2.33 (1.21, 4.51)	0.012	1.79 (0.96, 3.33)	0.065	3.33 (1.82, 6.09)	<0.001
Diabetes	0.95 (0.51, 1.79)	0.88	2.13 (1.28, 3.53)	0.003	1.68 (1.00, 2.82)	0.05
Hypertension	0.72 (0.40, 1.29)	0.27	1.24 (0.71, 2.15)	0.46	0.62 (0.36, 1.07)	0.087
Ischaemic heart disease	1.91 (0.80, 4.57)	0.15	1.07 (0.51, 2.26)	0.86	1.32 (0.62, 2.81)	0.47
Stroke	1.38 (0.27, 6.94)	0.7	1.23 (0.32, 4.69)	0.76	1.65 (0.43, 6.37)	0.47
Chronic kidney disease	0.37 (0.14, 0.98)	0.044	0.72 (0.36, 1.44)	0.36	0.59 (0.29, 1.18)	0.13
Polypharmacy	0.96 (0.38, 2.39)	0.93	1.33 (0.65, 2.73)	0.43	1.40 (0.67, 2.92)	0.36
Current smoker	0.69 (0.29, 1.64)	0.4	0.87 (0.43, 1.78)	0.71	1.08 (0.54, 2.16)	0.83
Alcohol frequency						
None	Reference		Reference		Reference	
≤4 days per week	0.54 (0.26, 1.12)	0.1	0.82 (0.42, 1.59)	0.56	0.69 (0.36, 1.32)	0.27
>4 days per week	2.22 (0.41, 11.80)	0.35	5.23 (1.12, 24.46)	0.036	2.86 (0.61, 13.33)	0.18
Low physical activity	1.16 (0.71, 1.90)	0.55	1.55 (1.01, 2.38)	0.043	1.76 (1.14, 2.71)	0.011
Low caloric intake	1.12 (0.67, 1.88)	0.66	0.86 (0.55, 1.35)	0.52	1.09 (0.69, 1.72)	0.71

Model was adjusted for age, sex, ethnicity, low socioeconomic status, living alone, high C‐reactive protein level, obesity, presence of systemic comorbidities (diabetes, hypertension, ischaemic heart disease, stroke, chronic kidney disease, and osteoporosis), polypharmacy, smoking status, weekly alcohol consumption, low physical activity, and low caloric intake.

CI, confidence interval; RRR, relative rate ratio.

### Factors associated with components of poor muscle health stratified by ethnicity

Ethnicity stratified analyses (Table 5) revealed differences in the determinants of poor muscle components; for example, in Chinese, most of the above factors remained significant except obesity (not associated with poor muscle function), diabetes and low PA (both not associated with pre‐sarcopenia); moreover, CKD was additionally associated with lower odds of all three components (low muscle function: RRR: 0.42, 95% CI: 0.16, 1.08; *P* = 0.073; pre‐sarcopenia: RRR: 0.33, 95% CI: 0.11, 1.05; *P* = 0.061; any sarcopenia: RRR: 0.35, 95% CI: 0.14, 0.89; *P* = 0.028). For Malays, only older age was a risk factor for any sarcopenia (per year increase: RRR: 1.17, 95% CI: 1.07, 1.27; *P* < 0.001), and obesity was protective of pre‐sarcopenia (RRR = 0.18, 95% CI: 0.05, 0.68; *P* = 0.012) and any sarcopenia (RRR = 0.17, 95% CI: 0.07, 0.43; *P* < 0.001). For Indians, only low PA was associated with higher likelihood of any sarcopenia (RRR: 2.74, 95% CI: 1.09, 6.89; *P* = 0.033); the presence of osteoporosis was risk factor for pre‐sarcopenia (RRR: 2.33, 95% CI: 1.21, 4.51; *P* = 0.012) and any sarcopenia (RRR: 2.33, 95% CI: 1.21, 4.51; *P* = 0.012) only; obesity was associated with 2.8 times (95% CI: 1.04, 7.52) the likelihood of poor muscle function but was protective of pre‐sarcopenia (RRR: 0.24, 95% CI: 0.07, 0.80; *P* = 0.02); moreover, hypertension was additionally associated with lower odds of all three components (RRR: 0.14, 95% CI: 0.02, 1.16; *P* = 0.068 for poor muscle function; RRR: 0.09, 95% CI: 0.01, 0.92; *P* = 0.043 for pre‐sarcopenia; RRR: 0.08, 95% CI: 0.01, 0.069; *P* = 0.022 for any sarcopenia).

**Table 5:** Factors associated with components of poor muscle health stratified by ethnicity

Risk factors	Pre‐sarcopenia RRR (95% CI)^a^			Poor muscle function RRR (95% CI)^a^			Any sarcopenia RRR (95% CI)^a^		
	Chinese	Malay	Indian	Chinese	Malay	Indian	Chinese	Malay	Indian
Age	1.05 (0.99, 1.12)	1.00 (0.88, 1.13)	1.07 (0.94, 1.20)	1.15* (1.08, 1.22)	1.07 (0.99, 1.15)	1.18* (1.07, 1.31)	1.26* (1.19, 1.34)	1.17* (1.07, 1.27)	1.23* (1.11, 1.37)
Sex (female)	2.72* (1.37, 5.41)	0.38 (0.09, 1.56)	0.76 (0.22, 2.59)	4.18* (2.11, 8.28)	1.58 (0.60, 4.13)	2.10 (0.77, 5.68)	2.33* (1.16, 4.30)	0.53 (0.19, 1.51)	0.96 (0.35, 2.66)
Living alone	1.89 (0.48, 7.51)	1.94 (0.10, 36.96)	0.70 (0.05, 9.74)	1.58 (0.41, 6.13)	2.41 (0.30, 19.61)	1.61 (0.18, 14.20)	1.66 (0.45, 6.22)	3.92 (0.44, 34.6)	1.39 (0.16, 12.37)
High C‐reactive protein	1.07 (0.48, 2.40)	0.82 (0.22, 3.09)	1.05 (0.31, 3.60)	1.40 (0.66, 2.97)	0.70 (0.32, 1.53)	1.10 (0.40, 3.02)	1.10 (0.52, 2.3)	1.06 (0.44, 2.54)	1.29 (0.46, 3.62)
Obesity	0.23* (0.10, 0.51)	0.18* (0.05, 0.68)	0.24* (0.07, 0.80)	1.67 (0.86, 3.25)	1.33 (0.56, 3.17)	2.80* (1.04, 7.52)	0.28* (0.14, 0.55)	0.17* (0.07, 0.43)	0.54 (0.20, 1.44)
Osteoporosis	2.23 (0.96, 5.17)	2.67 (0.57, 12.45)	6.06* (1.09, 33.82)	2.34* (1.03, 5.58)	1.41 (0.48, 4.12)	1.59 (0.31, 8.06)	4.66* (2.11, 10.30)	2.01 (0.66, 6.17)	4.74 (0.96, 23.27)
Diabetes	1.28 (0.50, 3.32)	0.95 (0.23, 3.94)	0.68 (0.21, 2.17)	4.06* (1.75, 9.43)	0.87 (0.36, 2.08)	1.77 (0.68, 4.61)	2.44* (1.05, 5.69)	1.00 (0.38, 2.61)	1.17 (0.44, 3.08)
Hypertension	0.98 (0.48, 2.00)	2.41 (0.55, 10.57)	0.09* (0.01, 0.92)	1.91 (0.86, 4.23)	2.12 (0.83, 5.45)	0.14 (0.02, 1.16)	0.80 (0.39, 1.62)	1.55 (0.55, 4.39)	0.08* (0.01, 0.69)
Ischaemic heart disease	1.45 (0.38, 5.54)	1.87 (0.19, 18.10)	2.81 (0.74, 10.68)	1.57 (0.47, 5.22)	1.32 (0.26, 6.66)	0.92 (0.28, 3.09)	1.24 (0.39, 3.99)	2.02 (0.37, 10.99)	1.54 (0.57, 5.07)
Chronic kidney disease	0.33 (0.11, 1.05)	0.19 (0.02, 2.12)	1.15 (0.08, 15.62)	0.42 (0.16, 1.08)	0.99 (0.31, 3.20)	2.54 (0.28, 23.09)	0.35* (0.14, 0.89)	0.67 (0.19, 2.30)	2.35 (0.26, 21.02)
Polypharmacy	0.67 (0.18, 2.55)	2.63 (0.36, 19.19)	0.76 (0.16, 3.62)	0.63 (0.20, 1.97)	3.12 (0.80, 12.27)	1.63 (0.44, 6.08)	1.15 (0.38, 3.53)	2.69 (0.63, 11.40)	1.25 (0.33, 4.70)
Current smoker	0.62 (0.20, 1.90)	0.24 (0.03, 1.82)	1.33 (0.24, 7.34)	0.82 (0.26, 2.54)	1.17 (0.33, 4.13)	0.49 (0.11, 2.28)	0.70 (0.25, 1.96)	1.52 (0.42, 5.50)	1.17 (0.27, 5.03)
Low physical activity	1.34 (0.71, 2.52)	1.24 (0.36, 4.24)	2.05 (0.69, 6.06)	1.18 (0.98, 3.36)	1.26 (0.58, 2.76)	1.91 (0.78, 4.68)	2.37* (1.31, 4.31)	1.18 (0.50, 2.79)	2.74* (1.09, 6.89)
Low caloric intake	1.26 (0.65, 2.42)	0.45 (0.11, 1.88)	1.92 (0.61, 6.06)	1.23 (0.65, 2.33)	0.92 (0.40, 2.14)	0.59 (0.23, 1.56)	1.09 (0.58, 2.04)	1.17 (0.47, 2.96)	1.35 (0.51, 3.57)

CI, confidence interval; RRR, relative rate ratio.

^a^ Models were adjusted for age, sex, living alone, high C‐reactive protein level, obesity, presence of systemic comorbidities (diabetes, hypertension, ischaemic heart disease, chronic kidney disease, osteoporosis), polypharmacy, smoking status, low physical activity, and low caloric intake. Low socioeconomic status, stroke, and alcohol were dropped from the model due to low cell count.

* *P*‐value <0.05.

## Discussion

In our large, contemporary, and population‐based study of multiethnic Singaporeans aged ≥60 years, almost 90% had poor muscle health, comprising approximately 28%, 17%, and 44% of poor muscle function, pre‐sarcopenia, and any sarcopenia, respectively. Several modifiable factors of poor muscle health and associated components include insufficient physical activity, osteoporosis, diabetes, and obesity. Importantly, we found ethnic‐specific differences in risk factors (low PA, osteoporosis, diabetes, obesity, hypertension, and CKD) associated with poor muscle health and related components. Our results support the need for large‐scale community‐based targeted intervention programmes that incorporate individualized diet, and progressive resistance exercise for early detection and management of poor muscle health and constituents in older adults, to mitigate the debilitating impact of this geriatric syndrome and maintain functional independence.

Our national census‐weighted prevalence of poor muscle health was approximately 90%, which is lower than that of 98% found in the Strengthening Health In Elderly through nutrition (SHIELD) study.^11^ For the constituents of muscle health, the SHIELD study reported higher prevalence of sarcopenia (68.9%; based on criteria from the updated consensus by the AWGS 2019^6^) and lower prevalence of poor muscle function (17.6%) and pre‐sarcopenia (4.3%) than our study. These differences could be attributed to the inclusion of older adults at risk of malnutrition in the SHIELD study because malnutrition in itself is a risk factor of low muscle mass and poor muscle health.^3^ In addition, our prevalence of any sarcopenia at 44% is substantially higher than that reported in the recent Yishun study (32.2%; based on criteria from the updated consensus by the AWGS 2019^6^) in Singapore.^17^ However, the Yishun study only recruited 297 older (aged >60 years) adults (44.5% participants were aged <60 years) via purposive sampling methods from the northeastern part, which may have regional differences inherent in socio‐economic status to the rest of Singapore.^18^. As such, findings from these two studies may not be generalizable to the community‐dwelling older adult population in Singapore, unlike our study. Nevertheless, these high prevalence figures suggest an urgent need for the development and implementation of population wide targeted interventions for poor muscle health including sarcopenia in community dwelling older Singaporean individuals. Although screening may not be required, assessment and documentation of baseline muscle health measures will still be important in order to enable a targeted approach and monitor response to interventions.

Our finding of older age as a risk factor of poor muscle health supports previous studies that have established loss of muscle mass and strength with aging.^19^ Similar to other studies, we observed that presence of diabetes was associated with a higher odds of poor muscle health and components, likely due to insulin resistance accelerating loss of muscle mass/strength.^20^ We also found osteoporosis was associated with poor muscle health/components. Indeed, several studies have suggested a bidirectional relationship between sarcopenia and osteoporosis, including a recent meta‐analysis of 56 pooled studies (796 914 participants), which indicated that sarcopenia was significantly associated with the higher risk of osteoporosis (OR = 3.06; 95% CI, 2.30 to 4.08 from a pooled analysis of 38 studies) and vice versa, that is, osteoporosis was significantly related to higher sarcopenia risk (OR = 2.63; 95% CI, 1.98–3.49 from a pooled analysis of 17 studies).^21^ These findings highlight the importance of sarcopenia screening for those at risk of osteoporosis, and vice versa, and reinforce the concept of ‘osteoporosis‐skeletal muscle reduction’, likely due to common causes of aging, malnutrition, smoking and physical inactivity in the afflicted individuals, as well as alteration in common biological pathways of cytokines, myokines, and osteokines.^22^

Furthermore, we found that obesity increased the risk of low muscle function most likely as a result of myosteatosis,^23^ where an increase in fatty infiltration of muscle tissues lead to a decrease in muscle quality as well as physical performance.^23^ Interestingly, obesity was associated with lower likelihood of poor muscle health, pre‐sarcopenia and any sarcopenia. Previous studies have reported a similar association between low body mass index (BMI) and sarcopenia in older adults living in the community^24^ and in nursing homes.^25^ This is likely a result of the non‐discriminatory nature of BMI in terms of body composition, and as a result, individuals with a higher BMI may have concurrent higher fat mass and higher lean mass at the same time compared with an individual with low BMI due to greater stature alone. Indeed, in our study, in univariate analysis, obesity (BMI ≥ 30) was associated with higher lean mass (β: 1.32; 95% CI: 0.96, 1.67) and higher fat mass (OR = 715.1; 95% CI: 100.2, 5105.1; *P* < 0.001). A recent study in Hong Kong investigating the relationship between obesity and sarcopenia in older adults also found a protective effect between obesity as defined by BMI and sarcopenia in both genders. However, when obesity was defined using percentage body fat (body fat mass divided by body weight), there was now a positive correlation between obesity and sarcopenia, suggesting that how obesity is defined is important in determining the direction of association with sarcopenia, and that body composition assessment may be more important than BMI alone in the determination of muscle health.^26^ The relationship between body mass and muscle health is complex. This is because a large body mass may not only have a higher amount of fat mass but also a corresponding higher amount of muscle mass when compared with another individual with very low body mass or severely underweight. At the same time, there can also be the variation where a large body mass consists predominantly of fat mass, low muscle mass and low muscle function (i.e., low muscle strength and/or low muscle performance) leading to the condition defined as sarcopenic obesity. The latest guidelines by the European Society of Clinical Nutrition and Metabolism (ESPEN) and the European Society for the Study of Obesity (EASO) recommend that the assessment for sarcopenic obesity should only be undertaken if there is presence of low muscle function, highlighting that some individuals may have a high fat mass but are not sarcopenic and do not have poor muscle health.^27^ This also highlights the current shift in research from only looking at body mass or fat mass in isolation, and the focus instead on ‘optimal body composition’, particularly in old and very old adults where having a higher body mass can be protective.^28^ In terms of public health, the recommendations by the ESPEN and EASO working group is that individuals above 70 who are overweight or fulfil obesity criteria should be screened for poor muscle function regularly to diagnose sarcopenic obesity. Further studies are needed to untangle the link between obesity and muscle health.

We also found ethnic‐specific differences in determinants (low PA, osteoporosis, diabetes, obesity, hypertension, and CKD) of poor muscle health components. The mechanism may be linked to lower fat mass index (Table 1), thereby contributing to lower lean muscle mass of Chinese compared with Malays and Indians.^29^ The presence of osteoporosis was a risk factor in Chinese and Indian (only for pre‐sarcopenia and sarcopenia) ethnicities, while diabetes had detrimental effect and CKD was associated with a lower risk only in Chinese individuals. This could likely be due to Chinese having lower fat mass index than Malays and Indians (Table 1), and we know that in individuals with weight loss due to calorie restrictions, approximately 30% of total weight will likely also include loss of lean mass in obese or overweight individuals, and this loss of lean mass can exceed 35% in normal weight individuals.^29^ Similarly, low BMI is associated with both higher eGFR and poor bone health.^30^ Hence the findings with regards the strong link between the Chinese ethnicity with poor muscle health, diabetes, osteoporosis, and lower likelihood of CKD are likely due to very low lean mass in this ethnic group, as loss of muscle mass is associated with the increase in insulin resistance, poor bone integrity, increased risk of fractures and the known inverse relationship between BMI and eGFR.^30^, ^31^, ^32^ This is in contrast with the muscle wasting seen in advanced CKD as a result of the inflammation related protein energy wasting (PEW) state with the prevalence of sarcopenia increasing in tandem with progression of the severity of the advanced CKD.^33^ Additionally, these differences could be attributed to genetic predisposition but unlikely to be due to environmental influences, for example, differences in income, education levels and access to healthcare as these differences remained even after accounting for socioeconomic disparities. Differences in healthcare access between the three ethnic groups are also unlikely given the ready availability of subsidized healthcare across this small island‐country, although we cannot discount the impact of cultural differences (e.g., differences in health‐seeking behaviour),^13^ potentially contributing to these disparate ethnic‐specific muscle health determinant profile. As such, further cohort and experimental studies are needed to better understand the mechanisms underpinning these ethnic‐specific differences in muscle health determinant profiles observed in our study.

Although the reasons for the underlying relationship between the association of hypertension and lower odds of poor muscle health in the Indian participants are not clear, there have been reports on the very high consumption of salt in the diet of the Indian population in India ranging from 8 to 11 g per day, about twice the recommended 5 g per day by the World Health Organization.^34^ Although much of the dietary salt intake was reported to be added on during meal preparation, the main sources of salt in the diet were from meat, poultry, eggs, followed by dairy and dairy products, followed by fish and seafood.^34^ In addition, a large study of Indian vegetarian diets from four geographical regions reported that Indian vegetarians consumed more carbohydrates, less protein and less salt in their diet as compared with non‐vegetarians. Vegetarians were also less likely to take part in physical activity.^35^ We do not have data on the amount of salt intake and the percentage of Indian participants who are vegetarians, but we hypothesize that the relationship between hypertension and muscle health are dietary driven. Muscle protein synthesis in older adults is sensitive to amount of dietary protein intake as a result of age‐related anabolic resistance.^36^ Hence, a higher dietary protein intake will lead to higher levels of muscle protein synthesis up to a threshold and may provide an explanation for the observed link between better muscle health measures and hypertension, as a result of higher salt intake in Indian participants More studies are required to investigate and confirm these hypotheses.

Collectively our results indicate that poor muscle health is a significant concern in older Asians and emphasize the need to incorporate baseline muscle health measures in basic medical assessments for older individuals to detect poor muscle health. Moreover, poor muscle function occurs almost twice as frequently compared with low muscle mass alone in our study, suggesting a focus on muscle function in community dwelling older adults is required. This is because loss of muscle strength may precede the development of overt muscle loss, a condition known as dynapenia.^7^, ^9^ Importantly, interventions to maintain healthy weight are potential strategies to protect muscle health while extreme weight loss and low BMI should be avoided. Clinically, progression to sarcopenia can be prevented using lifestyle interventions like nutrition management and resistance exercise training to minimize the detrimental impact associated with these deleterious health states. Indeed, pilot randomized controlled trials (RCTs), have shown that lifestyle intervention programs that incorporates nutrition management, exercise and health education intervention can improve functional fitness (i.e., muscle mass, strength, and gait speed) in older adults with sarcopenia.^37^

Strengths of our study include its large, well‐characterized, geographically representative study sample; our objective measure of muscle health and constituents; and comprehensive multivariable adjustments for a range of relevant confounders. Our novel and comprehensive evaluation of muscle health and its components provided greater insight into the prevalence and associated factors of these conditions, to advance the understanding of poor muscle health in Asian older adults. However, there are some limitations to acknowledge. First, as this was a cross‐sectional analysis, we cannot make causal inferences. For instance, our results cannot establish cause‐effect relationships between osteoporosis, CKD and poor muscle health and its components. Longitudinal data are needed to confirm the temporality and validity of these findings. We are currently collecting 4‐year follow‐up data for the PIONEER study, which will allow us to ascertain whether baseline muscle health is linked with incident adverse outcomes. Second, our definition of sarcopenia used cut‐offs for low muscle mass that were derived from 11 studies performed in East Asian countries (Korea, Japan, Taiwan, and China) and 1 study in Thailand.^6^ Although these AWGS cut‐offs have been used widely (including the latest AWGS consensus update 2019) and cited regularly, they may have limited generalizability due to lack of *data* from other Asian countries in South Asia, West Asia and South East Asia. Studies to provide normative data for lean muscle mass and other measures of muscle health in young adult populations in these countries are urgently required. Third, although DXA is the recommended method to measure muscle mass by the AWGS and is recommended as a reference standard as it is widely used, precise (precision error 1.2% for lean mass), feasible, safe and has low cost,^6^ it is important to note that it is still a surrogate measure of muscle mass as it includes other soft tissues in addition to muscle mass, and hence is not as accurate as other measures such as CT, MRI or Deuterium‐labelled creatine.^38^ Future studies in Asian populations are warranted to investigate more precise techniques that are also safe and clinically feasible, such as Deuterium‐labelled creatine, to accurately quantify muscle mass.^39^ Fourth, certain data, such as participants' medical history and history of falls, were self‐reported as we were unable to access electronic medical records for verification due to Singapore's strict data protection laws. Studies with objectively assessed medical conditions are needed to elicit a better understanding of the muscle health. Lastly, although the reported global prevalence of sarcopenia in adults is reported to range from 10% to 27% in a recent meta‐analysis,^40^ our study has found a significantly higher prevalence of poor muscle health which is consistent with other recent local studies in Singapore,^11^ suggesting that there may be a large underlying at risk population, particularly in older adults and older adults at risk of malnutrition, whom may progress to overt sarcopenia in the absence of early targeted intervention. Future longitudinal studies will be required to confirm this, and the value of poor muscle health as a target in terms of primary prevention efforts for sarcopenia.

In conclusion, 9 in 10 community‐dwelling Singaporean individuals over 60 years had poor muscle health. With this great burden in mind, we reported several modifiable factors such as low physical activity and diabetes, osteoporosis, and obesity. Importantly, there were ethnic disparities in the factors associated with poor muscle health. Our results suggest targeted multimodal intervention programs for muscle health are needed urgently. These programs should incorporate individualized nutrition management and progressive resistance exercise for older adults diagnosed with poor muscle health to prevent the grave impact of poor muscle health and promote healthy aging.^5^

## Conflict of Interest

All authors have no conflict of interest.

## Funding

Professor Lamoureux is supported by the National Medical Research Council Senior Clinician Scientist Award (NMRC‐CSA‐SI #JRNMRR140601). The grant body had no roles in design, conduct or data analysis of the study, nor in manuscript preparation and approval.

## Supporting information


**Data S1.** Supporting Information

## Data Availability

All data generated or analysed during this study are included in this article.
