# Correction to “The Prevalence Patterns and Risk Factor Profiles of Poor Muscle Health and Its Associated Components in Multi‐Ethnic Older Asians: The PIONEER Study”

**DOI:** 10.1002/jcsm.13774

**Published:** 2025-03-27

Gupta P , Vu TA , Man REK , et al., The prevalence patterns and risk factor profiles of poor muscle health and its associated components in multiethnic older Asians: The PIONEER study. Journal of cachexia, sarcopenia and muscle. Aug 2024;15(4):1376–1387. doi:10.1002/jcsm.13483 38646827 PMC11294041

In the Funding ‘section’, we would like to add: “This research is supported by the National Medical Research Council (NMRC) through the SingHealth PULSES II Centre Grant (CG21APR1013).” After this sentence, it continues to read: “The grant body had no roles in design, conduct or data analysis of the study, nor in manuscript preparation and approval.”

We apologize for this error.

