# Supplementary material for: The prevalence patterns and risk factor profiles of poor muscle health and its associated components in multiethnic older Asians: The PIONEER study
Source: J Cachexia Sarcopenia Muscle. 2024 Apr 22;15(4):1376–87. doi: 10.1002/jcsm.13483 (PMC11294041; doi:10.1002/jcsm.13483)
Supplement: Supplementary file 1 — Data S1. Supporting Information [file JCSM-15-1376-s001.docx]

**Supplementary Material**

**Methods**

**Study Population and Design**

In brief, 6,377 individuals were selected using an age-, sex-, and ethnicity- stratified sampling framework from a national database. Malays, Indians, females, and older age groups were deliberately oversampled to ensure adequate precision of estimates during statistical analyses. Study invitation letters were then sent out in batches, sorted by residential addresses; followed by home visits from study recruitment officers to ascertain an individual’s eligibility and agreement to participate. Of the invited individuals, 1,015 (15.9%) were classified as ‘uncontactable’ because of invalid address(s); were unresponsive to ≥3 home visit attempts and/or our recruitment officers were unable to access their residence(s) because of security restrictions. In addition, 648 (10.2%) individuals were excluded because they were incarcerated; were residing in nursing homes/outside Singapore; or were deceased; while a further 994 (15.6%) were deemed ineligible because they were terminally ill, bedridden, or otherwise unable to give informed consent due to severe cognitive or hearing impairment or muteness. Of the remaining 3,720 (69.4%) eligible individuals, 2,643 (71.1%) took part in the study, 1,054 (28.3%) refused, and 22 (0.6%) were undecided (71.5% response rate). Reasons for refusal included lack of interest (n=895, 84.9%) or time needed to participate in the study (n=159, 15.1%). Compared to participants (n=2,643), non-participants (n=1,054) were older (p<0.001), more likely to be female (p<0.001), and more likely to be Chinese (p<0.001; all data not shown).

**Assessment of Covariables, Risk Factors, and Associated Definitions**

Participants’ sociodemographic details, including age, sex, ethnicity, socio-economic status (SES) (comprising income, education, and housing type); self-reported medical history (presence of diabetes, hypertension, hyperlipidemia, ischemic heart disease, and stroke; number of medications); and lifestyle factors (smoking status and frequency of alcohol consumption) were collected via an in-house questionnaire. *Low SES* was defined as having primary or lower education, and household monthly income < SGD$2000, and residing in a 1-2 room public housing flat. *Polypharmacy* was defined as taking ≥ 5 chronic disease medications (excluding short-term medication, e.g., pain; supplements; or vitamins).(1) *Nutrition* (dietary) information was collected via an electronic Food Frequency Questionnaire (FFQ), developed and validated in the local multi-ethnic population in Singapore.(2) Participants’ weekly food intake was categorized into 146 separate food groups and the corresponding caloric values for protein, fat and carbohydrate consumption were extracted for analyses. Physical Activity (PA) was assessed based on self-reported time (in hours) spent doing light (e.g., office work, driving a car, strolling), and moderate-vigorous activities (e.g., gardening, brisk walking, dancing, jogging). *Low PA* levels were defined as the sex-specific lowest quintile of total self-reported duration spent carrying out moderate and vigorous activity (e.g., gardening, brisk walking, dancing, jogging).

Clinical covariates were obtained via a standardized clinical examination. Two measurements of systolic blood pressure (SBP) and diastolic blood pressure (DBP) were taken using a digital automatic BP monitor (Dinamap Pro Series DP110X-RW; GE Medical Systems Information Technologies, Inc), and a third measurement was obtained if the 2 previous SBP or DBP readings differed by more than 10- or 5-mm Hg, respectively. The mean of the two closest measurements was used in analyses. *Hypertension* was defined as SBP ≥140mmHg, DBP ≥90mmHg, self-reported use of antihypertensive medications, or self-reported history of physician-diagnosed hypertension. BMD was classified according to the WHO criteria that are based on DXA measured T-scores of the lumbar spine, and/or femoral neck, and/or total hip as follows: normal (T-score>-1 SD), osteopenia (-1≥T-score>-2.5 SD), and *osteoporosis* (T-score≤-2.5SD).(3) *Fat mass index* (*FMI*) was calculated by dividing the individual’s total body fat mass (kg), as measured by DXA, by their height (m) squared. The recent Yishun study conducted in Singapore found FMI as the most preferred measure for adiposity. Thus, we defined obesity as FMI > 7.63kg/m^2^ for men and > 9.93kg/m^2^ for women, based on the sex-specific upper two quintiles of the Yishun study population.(4)

Blood samples were collected for hemoglobin A1c (HbA1c), random glucose, and total, high-density lipoprotein, low-density lipoprotein cholesterol, triglycerides, and serum creatinine measurements. *Inflammation* was defined as high C-reactive Protein (CRP) > 2 mg/L; *Diabetes* was defined as random glucose ≥11.1mmol/L, HbA1c ≥6.5%, self-reported use of diabetic medication or reported history of physician-diagnosed diabetes. *Hyperlipidemia* was defined as high levels of total cholesterol (≥ 6.2 mmol/L) and/or self-reported use of lipid-lowering medications. *Ischemic heart disease* (IHD) and stroke was defined as self-reported history of myocardial infarction or angina, and stroke, respectively, similar to previous epidemiologic studies conducted by our group.(5) *Chronic kidney disease* (CKD) was defined as an estimated glomerular filtration rate <60ml/min/1.73 m^2^.(6)

**REFERENCES**

1. Halli-Tierney A, Scarbrough C, Carroll DG. Polypharmacy: evaluating risks and deprescribing. American family physician. 2019;100(1):32-8.

2. Neelakantan N, Whitton C, Seah S, Koh H, Rebello SA, Lim JY, et al. Development of a Semi-Quantitative Food Frequency Questionnaire to Assess the Dietary Intake of a Multi-Ethnic Urban Asian Population. Nutrients. 2016;8(9).

3. Kanis JA. Assessment of fracture risk and its application to screening for postmenopausal osteoporosis: synopsis of a WHO report. WHO Study Group. Osteoporosis international : a journal established as result of cooperation between the European Foundation for Osteoporosis and the National Osteoporosis Foundation of the USA. 1994;4(6):368-81.

4. Pang BWJ, Wee SL, Lau LK, Jabbar KA, Seah WT, Ng DHM, et al. Obesity Measures and Definitions of Sarcopenic Obesity in Singaporean Adults - the Yishun Study. The Journal of frailty & aging. 2021;10(3):202-10.

5. Wong MYZ, Man REK, Gupta P, Lim SH, Lim B, Tham YC, et al. Is Corneal Arcus Independently Associated With Incident Cardiovascular Disease in Asians? American journal of ophthalmology. 2017;183:99-106.

6. Levey AS, Stevens LA, Schmid CH, Zhang YL, Castro AF, 3rd, Feldman HI, et al. A new equation to estimate glomerular filtration rate. Ann Intern Med. 2009;150(9):604-12.

7. Cava E, Yeat NC, Mittendorfer B. Preserving Healthy Muscle during Weight Loss. Adv Nutr. 2017;8(3):511-9.

8. Batsis JA, Villareal DT. Sarcopenic obesity in older adults: aetiology, epidemiology and treatment strategies. Nature reviews Endocrinology. 2018;14(9):513-37.

9. Han S, Park J, Nah S, Jang HD, Han K, Hong JY. Severity of underweight and risk of fracture: a Korean nationwide population-based cohort study. Scientific reports. 2022;12(1):10153.

10. Marcotte-Chénard A, Oliveira B, Little JP, Candow DG. Sarcopenia and type 2 diabetes: Pathophysiology and potential therapeutic lifestyle interventions. Diabetes & metabolic syndrome. 2023;17(9):102835.

11. Kirk B, Zanker J, Duque G. Osteosarcopenia: epidemiology, diagnosis, and treatment-facts and numbers. Journal of cachexia, sarcopenia and muscle. 2020;11(3):609-18.

12. Kawamoto R, Kohara K, Tabara Y, Miki T, Ohtsuka N, Kusunoki T, et al. An association between body mass index and estimated glomerular filtration rate. Hypertension research : official journal of the Japanese Society of Hypertension. 2008;31(8):1559-64.

13. Lim ASH, Bishop GD. The role of attitudes and beliefs in differential health care utilisation among Chinese in Singapore. Psychol Health. 2000;14(6):965-77.
